# Which Are the Most Burdensome Functioning Areas in Depression? A Cross-National Study

**DOI:** 10.3389/fpsyg.2016.01342

**Published:** 2016-08-31

**Authors:** Kaloyan Kamenov, Francisco Felix Caballero, Marta Miret, Matilde Leonardi, Päivi Sainio, Beata Tobiasz-Adamczyk, Josep Maria Haro, Somnath Chatterji, José Luis Ayuso-Mateos, Maria Cabello

**Affiliations:** ^1^Centro Investigación Biomédica en Red, Instituto de Salud Carlos IIIMadrid, Spain; ^2^Department of Psychiatry, Universidad Autónoma de MadridMadrid, Spain; ^3^Instituto de Investigación Sanitaria del Hospital de La Princesa, Hospital Universitario de la PrincesaMadrid, Spain; ^4^Neurology, Public Health and Disability Unit, Neurological Institute “C. Besta” IRCCS FoundationMilan, Italy; ^5^National Institute for Health and WelfareHelsinki, Finland; ^6^Department of Medical Sociology, Jagiellonian University Medical CollegeKrakow, Poland; ^7^Centro de Investigación Biomédica en Red de Salud Mental, Parc Sanitari Sant Joan de DéuBarcelona, Spain; ^8^Department of Health Statistics and Information Systems, World Health OrganizationGeneva, Switzerland

**Keywords:** functioning, disability, quality of life, depression, cross-national study

## Abstract

**Background:** The study aimed to identify the most burdensome functioning domains in depression and their differential impact on the quality of life (QoL) of individuals from nine countries in Asia, Africa, Europe, and Latin America.

**Materials and Methods:** Data from two multi-country projects—the World Health Organization’s Study on Global Ageing and Adult Health (SAGE) and the Collaborative Research on Ageing in Europe (COURAGE)—were analyzed. Eight functioning domains (pain, mobility, self-care, cognition, interpersonal activities, domestic life, and work, sleep and energy, and affect) and QoL were assessed in 4051 individuals with depression.

**Results:** The analyses of the pooled sample showed that *affect* (ß = –0.21, *p* < 0.001), *domestic life and work* (ß = –0.16, *p* < 0.001) and *interpersonal activities* (ß = –0.15, *p* < 0.001) were the most affected functioning domains. When the analysis was stratified by gender, women showed similar patterns to the total sample, whereas *mobility*, *self-care*, *cognition* and *pain* were not significant amongst men. The cross-national analysis revealed that difficulties in *affect* and *interpersonal activities* were common across countries, whereas the rest of the domains showed country variability. In addition, being a woman (ß = –0.05), being older (ß = 0.07), being married (ß = 0.05), not having a comorbid condition (ß = –0.03) and having a higher education (ß = 0.04) were all factors associated with higher levels of QoL.

**Conclusion:** There was a variation in the level of decrements in different functioning domains across countries. This is in line with the growing evidence that reporting functioning sum-scores obscures potential differences among people. Functioning tools should capture the distinctiveness among individuals in order to provide tailored responses.

## Introduction

In spite of all evidence based treatments, depression still represents a huge burden to society (Chisholm et al., 2004) due to the disability it causes (Ferrari et al., 2013), its high mortality rates (Cuijpers and Schoevers, 2004), suicide risk (World Health Organization, 2012), and economic impact (Sobocki et al., 2006). The diagnosis of depression is based on a number of symptomatic criteria (World Health Organization, 1992; American Psychiatric Association, 2000, 2013), but it has been argued that this broad diagnosis might lump individuals suffering from different syndromes into one category (Fried and Nesse, 2014). Therefore, there is a growing body of literature suggesting that the depressive symptoms need to be analyzed individually (Lux and Kendler, 2010), shifting the focus from assessing not only the number but also their nature (Lux and Kendler, 2010). Fried and Nesse (2014) analyzed the concurrent effects of individual depressive symptoms on the functional state of depressed individuals and found that the symptoms vary substantially in their associations with impairment. Sad mood and concentration problems were found to be the most debilitating symptoms.

There is also a growing recognition of the importance of improving functioning when treating depression; in fact, normalization of a patient’s overall functioning is considered an important criterion for remission (Zimmerman et al., 2006a,b). However, more than 80% of interventional studies published in the last decade have reported only sum-scores of the instruments assessing functioning – e.g., Social Adjustment Scale (Weissman et al., 1978), Sheehan Disability Scale (Sheehan, 1983), WHODAS (Ustun et al., 2010), rather than domain-specific information (Kamenov et al., 2015). These sum-scores do not provide information on the differential impact of each functioning domain on the overall state of a person with depression. Thus, a higher score might indicate either a large number of mildly affected functioning areas or a few domains with marked decrements. It remains unclear whether distinct areas of functioning weight differently in terms of individual burden. The International Classification of Functioning, Disability and Health (ICF) Research Branch of the World Health Organization (WHO) developed an evidence-based Core Set for depression (Cieza et al., 2004) to address the broad spectrum of disability and functioning in depression. Until now the Core Set has not been sufficiently implemented in research studies, perhaps due to the complexity of the model or the large number of categories selected (Alvarezz, 2012). Cieza et al. (2014) proposed a minimal generic set of functioning domains, based on the ICF model, which reflects the experience of individuals with regard to their global health, but the article did not provide any disease-specific information related to depression.

Therefore, the first objective of this study was to identify the most burdensome domains of functioning according to their impact on the quality of life (QoL) of patients with depression. QoL was chosen as an indicator of the overall condition of depressed individuals because it refers to one’s satisfaction with life activities and one’s appraisal of life in general (IsHak et al., 2002). Moreover, QoL improvement and restoration have been considered the ultimate barometer of treatment success in depression (IsHak et al., 2011).

Furthermore, many studies found cross-national differences in the prevalence and symptom profiles of depression. A population-based study with more than 38000 participants from 10 countries revealed remarkable differences in the lifetime prevalence of depression ranging from 1.5/100 adults in Taiwan to 19.0/100 adults in Beirut (Weissman et al., 1996). The WHO Psychological Problems in General Health Care (PPGHC) study further showed a 15-fold variation in major depression prevalence, from lowest prevalence in Japan and China to highest prevalence in Brazil and Chile (Simon et al., 2002). On the other hand, cross-national differences in symptom profiles were also identified. Loss of energy, insomnia, concentration difficulties and thoughts of death appeared in all countries, whereas weight loss, increased appetite, hypersomnia, retardation, agitation and decrease in sexual interest were determined as country-specific (Weissman et al., 1996). A study by Waza et al. (1999) found that Japanese depressed patients experienced more and different somatic symptoms than American patients. Despite the rich evidence on prevalence and symptom differences in depression, little has been done in regard to potential cross-national differences in functional impairment. Studies have predominantly assessed functioning as a general concept rather than domain-specific. A study by Bromet et al. (2011) based on the World Mental Health survey found that the association between prevalence of depression and functional impairment was positive across high- and low-income countries. Moreover, Simon et al. (2002) showed that depression was universally associated with disability across countries with low, middle, and high prevalence rates of depression.

It is still unknown, however, whether there are cross-national differences in the level of impairment of individual functioning areas in depression. Such information could shed light on the distinctiveness among individuals across countries in order to facilitate the assessment of disability and provide tailored responses. Therefore, the second objective of this paper was to examine the cross-national diversity of the relationship between the functioning domains and QoL of depressed patients in nine countries: Finland, Poland, Spain, China, Ghana, India, Mexico, Russian Federation, and South Africa.

## Materials and Methods

### Sample and Procedure

The study used data obtained from the WHO’s Study on Global Ageing and Adult Health (SAGE) and the Collaborative Research on Ageing in Europe (COURAGE in Europe), two multi-country projects conducted between 2007 and 2012. The COURAGE survey was conducted in Finland, Poland, and Spain, whereas the SAGE survey was undertaken in China, Ghana, India, Mexico, the Russian Federation, and South Africa. The selected countries represent different geographical locations and levels of socio-economic and demographic status. Both projects collected data on participants aged 18+ years, with an emphasis on people aged 50+ years, from nationally representative samples. The details of the design and methods for Courage in Europe and SAGE are published elsewhere (Kowal et al., 2012; Leonardi et al., 2014).

The sample comprised 4051 non-institutionalized adults (18+) who were currently experiencing an episode of depression from China (296), Ghana (428), India (1522), Mexico (291), Russia (321), South Africa (168), Poland (288), Finland (136) and Spain (601). Trained lay interviewers undertook the face-to-face interviews at the respondents’ homes. The interviews in Mexico, Poland, Finland and Spain were completed by computer-assisted personal interview (CAPI), whereas in the remaining countries the researchers used a paper and pencil interview (PAPI). China was the only country where both CAPI and PAPI were used. Quality control procedures were undertaken during the fieldwork (Üstun et al., 2005). Informed consent from all participants was obtained. Ethical approval was obtained from the WHO Ethical Review Committee for SAGE and by Neurological Institute Besta for COURAGE, and by all local ethics research review boards (Helsinki and Uusimaa Hospital District, Finland; Jagiellonian University Medical College, Krakow, Poland; Parc Sanitari Sant Joan de Déu, Barcelona, Spain; La Princesa University Hospital, Madrid, Spain; Shanghai Municipal Centre for Disease Control and Prevention, Shanghai, China; Ghana Medical School, Accra, Ghana; International Institute of Population Sciences, Mumbai, India; National Institute of Public Health, Cuernavaca, Mexico; School of Preventive and Social Medicine, Russian Academy of Medical Sciences, Moscow, Russia; and Human Sciences Research Council, Pretoria, South Africa). The individual response rate ranged from 53% in Finland and Mexico to 93% in China.

### Measures

The process of translation and adaptation of instruments aimed to achieve conceptually equivalent versions of all instruments in each of the countries. The first step was a forward translation, where a health professional familiar with the terminology of the specific area translated the instruments from English to the particular language following series of instructions. Then, a bilingual expert panel including health experts and experts with experience in instrument development and translation revised the translation. The third step was a back-translation by an independent translator. Finally, a pre-testing on a target population representative of those who will be administered the questionnaire was done. The whole process was based on a method previously refined in the course of several WHO studies (World Health Organization, 2009).

#### Depression

Participants who had been diagnosed with depression by a physician and had been receiving treatment during the last 12 months were included in the study. In addition, since there are many cases of undiagnosed persons who actually experience depressive episodes (Sheehan, 2004; Volicer et al., 2011), we assessed depression with a set of symptomatic questions derived from the World Mental Health Survey version of the Composite International Diagnostic Interview (CIDI) for depression (Kessler and Ustun, 2004). The individual items were included in a diagnostic algorithm generating a diagnosis of “depressive episode” according to the criteria specified in the *International Statistical Classification of Diseases and Related Health Problems, 10th revision, Diagnostic Criteria for Research* (World Health Organization, 1992).

#### Functioning Domains

Functioning was assessed with a multi-domain measure (Salomon et al., 2003) that was developed as an answer to the WHO statement: “functioning and functioning domains constitute the operationalization that best captures our intuitive notion of health” (Cieza et al., 2014). The measure had been previously used in 70 countries from the World Health Survey (Salomon et al., 2003). Participants were asked about the difficulties they had experienced in the last 30 days in each of these domains. The responses to each question were recorded on a 5-point scale ranging from 1 (no difficulty/problem) to 5 (extreme difficulty/inability).

#### Mobility

The domain of mobility assessed the ability of a person to move and get around. The participants were asked whether in the last 30 days they have had any difficulty in “*vigorous activities*,” “*walking a long distance such as a kilometer (or equivalent)*,” “*walking 100 meters*,” “*moving around inside your home*,” “*getting out of your home*,” “*getting where you want to go, using private or public transport if needed*,” “*stooping, kneeling or crouching*,” “*standing up from sitting down*,” “*getting up from lying down*,” “*sitting for long periods*,” “*standing for long periods such as 30 min*,” “*picking up things with your fingers (such as picking up a coin from a table)*,” “*extending your arms above your shoulder level*” and “*carrying things (such as carrying grocery bags, water bottles, etc.).*”

#### Self-Care

This domain measured the capacity of a person to perform self-care activities by answering the following items: “*Overall, in the last 30 days, how much difficulty did you have in: ‘taking care of and maintaining your general appearance (for example, grooming, looking neat and tidy)*,’ ‘*staying by yourself for a few days*,’ ‘*washing your whole body*,’ ‘*getting dressed*,’ ‘*getting to and using the toilet*,’ ‘*eating (including cutting up your food)*.”

#### Cognition

This domain measured communication and thinking activities. The two items included *“Concentrating on doing something for 10 min*” and “*learning a new task, for example, learning how to get to a new place.*”

#### Pain

Pain was assessed with two items: “*How much of bodily aches or pain did you have?*” and “*how much difficulty did you have in your daily life because of your pain?”*

#### Interpersonal Activities

This domain assessed the ability of individuals to interact with other people through five items: “*Joining in community activities (for example, festivities, religious or other activities) in the same way as anyone else can*,” “*personal relationships or participation in the community*,” “*dealing with conflicts and tensions with others*,” “*making new friendships*” and “*dealing with people you do not know.*”

#### Domestic Life and Work

This domain assessed difficulties with day-to-day activities, such as *“taking care of your household responsibilities”* and *“your day-to-day work/school.”*

#### Sleep and Energy

This domain assessed difficulties in sleep patterns (“*sleeping, such as falling asleep, waking up frequently during the night or waking up too early in the morning*”) and energy level (“*not feeling rested and refreshed during the day (for example feeling tired, not having energy).*”

#### Affect

This domain assessed the emotional functioning of participants. The domain included four items: “*feeling sad, low or depressed*,” “*worry or anxiety*,” “*emotionally affected by your health problems*” and “*how much did these difficulties interfere with your life.*”

#### Quality of Life

Quality of life was assessed using a short version of the WHO Quality of Life (WHOQOL) instrument (Power, 2003; Skevington et al., 2004). This questionnaire has shown good cross-cultural validity (da Rocha et al., 2012). It was designed as a short and concise instrument of eight items reporting four domains—psychological, social, physical, and environmental, each assessed by two items. The overall QoL score (ranging between 0 and 100) was formed by the sum of the scores of the eight items, with higher scores indicating better quality of life.

#### Control Variables

Country, age, sex, marital status, presence of comorbid physical chronic conditions (angina, hypertension, asthma, arthritis, or diabetes) and level of education were included in the analysis as control variables.

### Statistical Analysis

Descriptive statistics including summary of the socio- demographic data of the participants were obtained.

Factor scores for each functioning domain were obtained and then the scores were transformed into a scale ranging from 0 to 100, with lower scores representing better functioning. Before calculating the factor scores for each of the domains of functioning, confirmatory factor analyses (CFA) were performed to assess the goodness-of-fit of the domains.

To examine the independent contributions of all domains of functioning on QoL, a multiple regression analysis was conducted. Age, sex, country, marital status, comorbidity, and educational level were introduced as covariates to control for potential confounders. In addition, the analysis was carried out on the entire sample of depressed individuals, on men and women and on each country separately. The independent variables were introduced simultaneously in the model because we examined the effect of different domains of functioning on QoL rather than introducing previously established models. The ordinary least squares (OLS) estimation was employed, since it has been shown to yield the best fit of data (Alonso et al., 2011). Standardized (β) coefficients indicated the level of association between the functioning domains and the covariates and QoL, since (β) can be applied as effect size in regression models. Since we conducted multivariate regression models, with several independent variables, the presence of multicollinearity was assessed by means of the Variance Inflation Factor (VIF). Values below 5 were considered adequate (Rogerson, 2001) and providing evidences for little or no multicollinearity in the data. STATA version 11.0 (Stata Corp, 2009) was used to analyze the socio-demographic data, to calculate the factor scores and to conduct the regression models. Amos version 22 (Arbuckle, 2013) was used for the CFA. Confidence intervals (CI) for hypothesis tests were constructed at the 95% confidence level.

## Results

### Characteristics of the Sample

Socio-demographic and clinical characteristics of the sample in each country are presented in **Table 1**. A total of 4051 people with depression took part in the study. The mean age of the total sample was 60 years (*SD* = 14.36). Women were the majority (66.3%) and 67% of the sample had not completed higher education. Differences could be seen in Russia and Finland, where more than 85% of the sample had completed secondary school. The mean QoL score was 53.59 (16.69).

**Table 1:** Characteristics of the population by country.

Characteristics	Spain	Poland	Finland	China	Ghana	Mexico	India	Russia	South Africa
	(601)	(288)	(136)	(296)	(428)	(291)	(1522)	(321)	(168)
Age mean (*SD*)	62.15 (14.52)	61.4 (15.9)	54.70 (16.03)	61.19 (10.72)	64.75 (12.54)	62.77 (13.12)	56.27 (14.72)	65.56 (12.65)	58.18 (10.28)
Women *n* (%)	434 (72.2)	198 (68.8)	103 (75.7)	191 (64.5)	254 (59.3)	227 (78)	954 (62.7)	244 (76)	107 (63.7)
Men *n* (%)	167 (27.8)	90 (31.3)	33 (24.3)	105 (35.5)	174 (40.7)	64 (22)	568 (37.3)	77 (24)	61 (36.3)
Current marital status *n* (%)									
In a partnership/married	297 (49.4)	120 (41.7)	60 (44.1)	228 (77)	192 (44.9)	179 (61.5)	1113 (73.1)	144 (44.9)	79 (47)
Not in a partnership	304 (50.6)	168 (58.3)	76 (55.9)	68 (23)	233 (54.4)	112 (38.5)	409 (26.9)	176 (54.8)	88 (52.4)
Education									
Less than secondary school *n* (%)	427 (71)	103 (35.8)	13 (9.6)	177 (59.8)	327 (76.4)	230 (79)	1272 (83.6)	45 (14)	127 (75.6)
Secondary school or higher level completed: *n* (%)	174 (29)	185 (64.2)	123 (90.4)	117 (39.5)	90 (21)	61 (21)	249 (16.4)	276 (86)	38 (22.6)
Quality of Life Mean (*SD*)	57.79 (17.42)	50.79 (17.9)	60.89 (17.6)	48.74 (16.98)	52.39 (16.30)	56.36 (14.68)	54.49 (15.28)	46.44 (17.71)	49.5 (16.71)

### The Impact of Functioning Areas on Quality of Life

Confirmatory factor analyses were performed to find evidence for unidimensionality and the use of a global score in each domain. In each case, a single-factor model was proposed considering the items assigned to the domain. Then the factor structure was tested. All functional areas presented an acceptable fit according to the goodness-of-fit indices: Comparative Fit Index (CFI) >0.90 and Root Mean Square Error of Approximation (RMSEA) <0.08 in all cases. For the domains that included only two items, Cronbach’s alpha value was calculated, being higher than 0.70. Inter-item correlation was also higher than 0.50 in each case, indicating a strong relationship between the two items and also providing evidence of unidimensionality.

The results from the multiple regression analysis (**Table 2**) revealed that *affect* (ß = –0.21, *p* < 0.001), *domestic life and work* (ß = –0.16, *p* < 0.001) and *interpersonal activities* (ß = –0.15, *p* < 0.001) were the most important functioning domains associated with QoL. *Sleep and energy*, *mobility* and *cognition* were also statistically significant, but their effect sizes were smaller compared to the former three. *Pain* (*p* = 0.14) and *self-care* (*p* = 0.86) were the only domains that were not statistically significant.

**Table 2:** Effect estimates of functioning domains on quality of life using multiple regression analysis.

Variables	Total			Women			Men		
	B (95% *CI*)	*p*	ß^∗^	B (95% *CI*)	*p*	ß	B (95% *CI*)	*p*	ß
Cognition	–0.04 (–0.07–0.01)	**0.001**	–0.07	–0.04 (–0.08–0.01)	**0.007**	–0.07	–0.04 (–0.08–0.01)	0.12	–0.06
Mobility	–0.05 (–0.09–0.01)	**0.007**	–0.08	–0.05 (–0.1–0.001)	**0.046**	–0.07	–0.06 (–0.13–0.01)	0.08	–0.09
Pain	–0.02 (–0.04–0.01)	0.14	–0.03	–0.03 (–0.05–0.004)	0.09	–0.04	0.01 (–0.04–0.04)	0.84	0.01
Self-care	–0.01 (–0.04–0.03)	0.86	0.01	0.02 (–0.02–0.07)	0.32	0.03	–0.07 (–0.13–0.01)	0.06	–0.08
Interpersonal act.	–0.11 (–0.14–0.08)	**<0.001**	–0.15	–0.13 (–0.16–0.10)	**<0.001**	–0.18	–0.07 (–0.12–0.03)	**0.003**	–0.11
Domestic life/Work	–0.09 (–0.12–0.06)	**<0.001**	–0.16	–0.10 (–0.13–0.06)	**<0.001**	–0.17	–0.08 (–0.12–0.03)	**0.001**	–0.13
Sleep and energy	–0.04 (–0.07–0.02)	**<0.001**	–0.07	–0.03 (–0.05–0.01)	0.06	–0.04	–0.08 (–0.11–0.04)	**<0.001**	–0.13
Affect	–0.15 (–0.18–0.12)	**<0.001**	–0.21	–0.17 (–0.20–0.13)	**<0.001**	–0.24	–0.11 (–0.16–0.06)	**<0.001**	–0.16
**Controlling variables**									
Sex	–1.75 (–2.75–0.75)	**0.001**	–0.05						
Age	0.09 (0.05–0.12)	**<0.001**	0.07	0.09 (0.04–0.14)	**<0.001**	0.08	0.08 (0.01–0.14)	**0.022**	0.06
Educational level	1.41 (0.26–2.56)	**0.017**	0.04	1.05 (–0.46–2.56)	0.17	0.03	1.98 (0.14–3.82)	**0.035**	0.06
Marital status	1.71 (0.71–2.71)	**0.001**	0.05	1.64 (0.42–2.85)	**0.008**	0.05	1.52 (–0.39–3.43)	0.12	0.04
Comorbidity	–1.46 (–2.47–0.45)	**0.005**	–0.03	–0.75 (–2.19–0.3)	0.14	0.03	–2.47 (–4.18–0.76)	**0.005**	–0.07

*Effect size, ß coefficient; Country was introduced as a covariate in the model. ^∗^ In bold, significant p-values at 95% confidence level.

When the analysis was separated by gender (**Table 2**), women showed similar patterns as the total sample, with the only difference being that *sleep and energy* was only marginally significant (ß = –0.04, *p* = 0.06). *Affect* remained the most important functioning area (ß = –0.24, *p* < 0.001), followed by *interpersonal activities, domestic life and work, mobility* and *cognition*. *Interpersonal activities* were more strongly associated with their QoL compared to the total sample. Men, however, showed different patterns compared to the total sample and women in particular. *Affect* was still the most fundamental factor, but *sleep and energy* (ß = –0.13, *p* < 0.001) appeared to be the second major functioning area for men as opposed to women. *Self-care* was only marginally significant (ß = –0.08, *p* = 0.06), whereas *cognition* (ß = –0.06, *p* = 0.12), *mobility* (ß = –0.09, *p* = 0.08), and *pain* (ß = 0.001, *p* = 0.84) were not statistically significant functioning domains in men.

Furthermore, with respect to the control variables, being a woman (*p* = 0.001), being older (*p* < 0.001), being married (*p* = 0.001), not having a comorbid condition (*p* = 0.005), having a higher education (*p* = 0.017) and living in a specific country (compared to China; *p* < 0.001) were associated with higher levels of QoL in people with depression. There were gender differences only in terms of educational level, comorbidity and marital status—the former two not being significant for women and the latter for men. All the variables considered in the analyses had an associated VIF value lower than 5, indicating that the assumption of no perfect multicollinearity can be assumed to conduct the regression model.

### Cross-National Differences in the Level of Impairment in Different Functioning Areas

**FIGURE 1:**
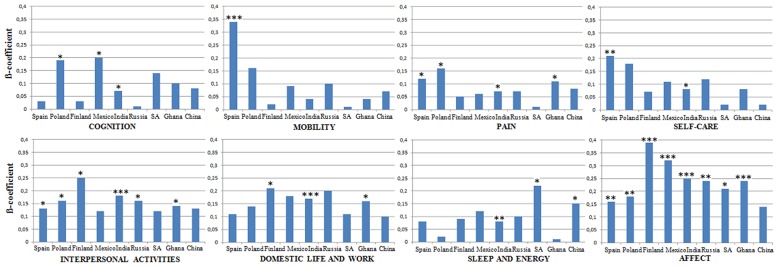
**Relationship between functioning domains and quality of life by country.** *^∗^p < 0.05, ^∗∗^p < 0.01, ^∗∗∗^p < 0.001.* Age, sex, marital status, comorbidity, and educational level were introduced as covariates to control for potential confounders. SA, South Africa.

We did a further cross-national analysis, controlling again for sex, age, marital status, level of education and comorbid conditions by exploring the most relevant functioning domains in each of the nine countries. *Affect* was significant in all countries except China. The second most commonly important domain was *interpersonal activities*, significant in all countries except Mexico, China and South Africa. *Pain* was significantly associated with QoL in Spain, Poland, India and Ghana. *Domestic life and work, cognition*, and *sleep and energy* showed statistical significance only in three of the nine countries. *Mobility* (ß = –0.34, *p* < 0.001) was the least represented domain across countries, being significant only in Spain. On the other hand, looking at the number of significant areas for each country, in India seven out of eight domains were significant (only *mobility* was not significant), followed by Spain with five (*interpersonal activities, mobility, pain, self-care, and affect*). China was the only country where only one functioning domain was found statistically significant – *sleep and energy* (ß = –0.15, *p* < 0.05). Detailed results for each country can be seen in **Figure 1**.

## Discussion

The present study examined the differential association of eight functioning domains on QoL in a large and representative sample of 4.051 depressed individuals across nine countries. Our findings suggest that *affect*, *interpersonal activities* and *domestic life and work* had the highest impact on QoL in depression. *Sleep and energy*, *mobility* and *cognition* were also important factors, but their effect was smaller. *Self-care* and *pain* were the only domains that were non-significant for men or women. A possible explanation might be that the participants showed relatively higher levels of self-care and lower levels of pain, and severe decrements in these domains were rare.

Only one study to date has attempted to identify a set of the most relevant functioning domains but does not provide anything disease-specific, but rather general clinical information (Cieza et al., 2014). The authors of the study suggested that *mobility, pain, sleep and energy*, and *affect* were the most important functioning domains associated with the general health of both clinical and general populations. Our study coincides with some of these findings, but the degree of importance varied. *Affect* was the most relevant domain associated with QoL, but *pain* was not significant in our sample. *Interpersonal activities*, however, which was the second most important domain in our study, was not considered in the final set of Cieza et al. (2014). This result is not surprising, as previous studies have already reported that people with depression experience significantly poorer intimate relationships and less satisfying social interactions than individuals with other psychiatric disorders, or the general population (Fredman et al., 1988).

We further examined differences across the included countries. *Affect* was significant in all countries except China. This supports previous evidence that emotional problems, such as low mood, are a core part of the experience of depression (Fried and Nesse, 2014) and chronic physical or mental conditions in general (Weigl et al., 2004). However, most of the generic functioning tools, such as WHODAS-II or SF-36 (Ware and Sherbourne, 1992), do not include affect as a single domain. Difficulties in *interpersonal activities* were also prevailing in most of the countries. However, the rest of the domains were country specific. *Cognition* was associated with QoL in Poland, India and Mexico; *mobility* in Spain; and *sleep and energy* in India, South Africa and China. In India we found a significant positive association between *pain* and QoL (ß = 0.07, *p* = 0.025) after controlling for sociodemographic variables and the remaining functioning domains. This finding is counterintuitive, as it indicates that a higher pain is associated with better QoL. In the rest of the countries where *pain* was associated with QoL— Spain, Poland and Ghana—we found a negative relationship between both. We examined the correlation coefficient between QoL and *pain* and found a significant value of *r* = –0.340 (*p* < 0.001). However, after controlling for *affect* we found a non-significant partial correlation coefficient. After we ran the previous regression model without considering *affect* as covariate we did not find any significant association between *pain* and QoL. This suggests that the relationship between *pain* and QoL in India was moderated by *affect*. Similarly, a counterintuitive association was found between *self-care* and QoL in Spain. We found a highly significant correlation between *self-care* and *mobility* in the Spanish sample (*r* = 0.764), which suggested potential multicollinearity in the model. After *mobility* was removed from the regression model, *self-care* was no longer significantly associated with QoL.

The reason why some functioning domains were associated with QoL only in some countries but not in others is unclear, but certain cultural contrasts might be responsible for these variations. For instance, in our study *mobility* was more important for the Spanish population than for any other country. Previous studies evaluating the health status in general population confirm this result, showing that Spanish raters place more importance on *mobility* as a functioning domain compared to other countries due to cultural or lifestyle characteristics (Badia and Alonso, 1995; Badia et al., 2001).

Even though many of the effect sizes associated with the results obtained from the regression models were small or moderate, the results of this study have considerable practical implications. Firstly, in a research perspective, our results boost future changes in the measurement of disability in depression. The study proves that there is a variation in the level of decrements in different functioning domains across countries. Furthermore, it provides a basis for further development of more sensitive, cross-nationally validated and user-friendly instruments weighting the domains according to their importance and providing a better picture on the living experience of depressed individuals. This action is urgent, given the fact that only 5–20% of all clinical trials for depression report functioning outcomes (McKnight and Kashdan, 2009; Kamenov et al., 2015) and to date there is no gold standard tool for measuring functioning in depression (Lam et al., 2015). Symptoms of depression, which are the main focus of clinical trials, provide early signs of treatment response, but the functional outcomes provide an indicator of meaningful change, thus making the inclusion of functional tools in clinical trials a pressing issue (McKnight and Kashdan, 2009). One reason for the lack of comprehensive data on functioning is the complex conceptualization of the term and the lack of disease-specific information on all relevant areas. Another explanation is that they lack comprehensiveness and cultural validity. Therefore, the Canadian Network for Mood and Anxiety Treatments (CANMAT) depicts as a primary aim the development of a scale for measuring functioning outcomes in clinical trials that could be used or adapted for different clinical care settings (Lam et al., 2015). Although several recent trials have attempted to apply newly developed measures designed to capture a more comprehensive array of functioning difficulties, all of them are still in their infancy, do not provide domain-specific information or have not been validated in cross-national samples (Cohen et al., 2013; Zimmerman et al., 2013).

Secondly, the study reveals particular gender differences in the functional impairment of depression. This is an important finding which needs further exploration in other cross-national samples, given the higher prevalence of depression in women compared to men. Different level of impairment in specific functioning areas might be a key to the understanding of these different prevalence rates. Thirdly, our study promotes the need of reporting domain-specific information in studies for better understanding the living experience of depression. Studies predominantly report only the sum-scores of the instruments. These sum-scores do not provide information on the differential impact of each functioning domain on the QoL of individuals. A higher sum-score might indicate either a large number of mildly affected functioning areas or a few domains with marked decrements. Potentially important information on functioning is lost, and a detailed analysis of these functioning domains is likely to reveal important information hidden by the sum-scores. Last, but not least, our study raises the question about the importance of assessing functioning in clinical trials and expanding the diagnostic criteria for depression. Gaining insight on the socio-culturally based differences in the areas of functioning in depression might be the key of promoting future culture-sensitive nosology accounting for functional impairment (Juhasz et al., 2012). At clinical level, with the development of a new instrument for functioning, clinicians experiencing time restraints should be given the opportunity to prioritize and meet the needs of patients promptly. Future studies following our line of research should be able to provide sufficient evidence for implementing national programs focusing on prevention and treatment of functional impairments in depression.

The present study has certain limitations. The data was cross-sectional, thus both QoL and functioning impairment were assessed at the same measurement point. However, our objective was to rank the importance rather than to explore temporal relations or infer causal relationships. Secondly, there were certain variations in the sample size of each country. For this study we selected only participants with depression, but both the COURAGE and SAGE samples were nationally representative. The differences in the number of depressed participants in each country might be due to differences in the prevalence of depression or cross-national differences in reporting in the self-reported instruments (Kessler and Bromet, 2013).

This is the first study to our knowledge to explore the differential impact of functioning domains on QoL in depression across different regions of the world. One of the strengths of the study is its large sample size and geographically and socio-economically diverse participants. Although more than 85% of the world’s population lives in low- and middle-income countries, most of the evidence comes from high-income countries (Saxena et al., 2006). Our paper, however, contributes with data not only from high-income countries, but also low and middle income countries. Our results showed that there was a variation in the importance of different functioning domains across countries. This is in line with the growing evidence that reporting functioning sum-scores blurs potential differences among people. The concealed variability within the concept of functioning has further led to disappointing findings—treatment in depression is only low-to-moderately efficacious for improving functioning (De Silva et al., 2013; Renner et al., 2014). Future research should focus on a more personalized approach to the assessment of functioning.

## Author Contributions

ML, PS, BT-A, SC, JH, MM, FC, and JA-M conceived, designed and performed the experiments; KK, FC, MC, and MM analyzed the data; KK, MC, MM, and FC wrote the paper; ML, PS, BT-A, SC, JH, JA-M, FC, MM, and MC made critical revisions of the manuscript for important intellectual content.

## Conflict of Interest Statement

The authors declare that the research was conducted in the absence of any commercial or financial relationships that could be construed as a potential conflict of interest.
